# Natural Killer Cell Dysfunction in Hepatocellular Carcinoma: Pathogenesis and Clinical Implications

**DOI:** 10.3390/ijms19113648

**Published:** 2018-11-19

**Authors:** Pil Soo Sung, Jeong Won Jang

**Affiliations:** 1 Department of Internal Medicine, College of Medicine, The Catholic University of Korea, Seoul 06591, Korea; pssung@catholic.ac.kr; 2 The Catholic Liver Research Center, College of Medicine, The Catholic University of Korea, Seoul 06591, Korea

**Keywords:** hepatocellular carcinoma, natural killer cell

## Abstract

Hepatocellular carcinoma (HCC) is currently the third leading cause of malignancy-related mortalities worldwide. Natural killer (NK) cells are involved in the critical role of first line immunological defense against cancer development. Defects in NK cell functions are recognized as important mechanisms for immune evasion of tumor cells. NK cell function appears to be attenuated in HCC, and many previous reports suggested that NK cells play a critical role in controlling HCC, suggesting that boosting the activity of dysfunctional NK cells can enhance tumor cell killing. However, the detailed mechanisms of NK cell dysfunction in tumor microenvironment of HCC remain largely unknown. A better understanding of the mechanisms of NK cell dysfunction in HCC will help in the NK cell-mediated eradication of cancer cells and prolong patient survival. In this review, we describe the various mechanisms underlying human NK cell dysfunction in HCC. Further, we summarize current advances in the approaches to enhance endogenous NK cell function and in adoptive NK cell therapies, to cure this difficult-to-treat cancer.

## 1. Introduction

Worldwide, hepatocellular carcinoma (HCC) is the third leading cause of cancer-related mortalities and the sixth most predominant type of malignancy [1]. The majority of patients with HCC are diagnosed at an advanced stage, contributing to the very low rate of five-year survival [2]. HCC often develops in patients with a history of chronic hepatitis B virus (HBV) and hepatitis C virus (HCV) infections [1]. In addition, obesity and diabetes, which cause non-alcoholic steatohepatitis (NASH), have become significant risk factors for the development of HCC in patients from developed countries, in recent years [1,3]. Antiviral therapies for HCV and HBV assist in reducing HCC occurrence [2], but the limitations of accessibility to these antivirals in developing countries and a rising incidence of NASH, guarantee that HCC is going to remain an impending perplexing disease [2].

It is challenging to treat patients with HCC in the advanced stage. The two drugs that FDA has approved for treating advanced HCC, as a first line therapy, are sorafenib and lenvatinib [4,5,6]. Nevertheless, only a modest survival improvement, as well as the substantial adverse effects, underscore the need for new therapeutics [5]. For sorafenib, the Sorafenib HCC Assessment Randomized Protocol (SHARP) trial showed that the drug only increased survival in patients with unresectable HCC from 7.9 months to 10.7 months [7]. Moreover, the therapeutic effects of sorafenib on extending patient survival were even less potent in Asian patients (from 4.2 months to 6.5 months) [8]. Lenvatinib was also proved to effective as a first-line treatment in advanced HCC, although the survival improvement was also modest [6]. In a recent phase III trial, a median overall survival was 13.6 months for lenvatinib and 12.3 months for sorafenib, indicating that lenvatinib was non-inferior to sorafenib in overall survival of patients with unresectable HCC [6]. After decades of failure of immune therapies, immune checkpoint inhibitors have been proven as effective treatments for patients with advanced HCC [9,10]. Monoclonal antibodies that are able to block immune checkpoint molecules displayed antitumor activity against HCC [10]. Clinical trials were performed with two checkpoint inhibitors in HCC: the anti-cytotoxic T lymphocyte-associated antigen 4 (CTLA-4) agent tremelimumab [11] as well as the anti-programmed death 1 (PD-1) agent nivolumab [12]. The latter has shown unprecedented antitumor activity in both sorafenib-naive and sorafenib-experienced patients with advanced HCC [12]. In a recent phase II trial, objective response rate of nivolumab in advanced HCC was 22.5% in sorafenib-naive and 18.7% in sorafenib-experienced patients [12]. Programmed death ligand-1 (PD-L1) expression of tumor cells and baseline serum alpha-fetoprotein (AFP) levels were not associated with tumor response to nivolumab [13]. These promising antitumor responses resulted in encouraging survival outcomes with a median overall survival of 28.6 months in sorafenib-naive and about 15 months in sorafenib-experienced patients [12]. However, the response rate of nivolumab is still below 20 percent [12]. Novel approaches that can harness antitumor immune response are therefore urgently required for HCC.

The liver contains a diverse population of innate lymphocytes, such as natural killer (NK) cells, natural killer T (NKT) cells, as well as adaptive lymphocytes like B cells and T cells [14]. The comparative description of immune cell subpopulations between human and mouse can help understand the human intrahepatic immune response more precisely. However, there are several differences when comparing human and mice immune cell subsets in the liver. More NK cells are contained in the human liver than in the mouse liver, although the frequency of Kupffer cells and dendritic cells (DCs) are similar in human and mouse liver [15]. Furthermore, instead of a relatively large proportion of the NKT cells in mouse liver, mucosal-associated invariant T cells (MAIT cells) predominate in human liver [15]. In this review, we will mainly focus on the human NK cells and related immune responses in the human liver.

Immune cells that reside in the liver are exposed to numerous antigens through the portal circulation and therefore, unfavorable immune responses are easy to be triggered [16,17]. It appears that the liver has attained specialized immune tolerance mechanisms to evade the undesirable immune over-activation. Experimental allogeneic multi-organ transplantation in pigs showed that liver allograft was significantly much better tolerated than other organs, demonstrating intrahepatic immune tolerance [18]. To maintain the tolerance, liver contains high numbers of immune regulatory cells [19]. Liver resident cells, including Kupffer cells, DCs, hepatic stellate cells, sinusoidal endothelial cells, and myeloid-derived suppressor cells (MDSCs) mediate immune tolerance by producing anti-inflammatory cytokines such as transforming growth factor-β (TGF-β) and interleukin-10 (IL-10), or by expressing inhibitory molecules for NK or T cell activation [19]. The development of chronic infectious disease by HCV or HBV, also appears to be related to the liver’s tolerogenic properties [20]. Virus-specific T cell responses are usually delayed and impaired in chronic HCV and HBV infection, reflecting the tolerogenic property of human liver [21]. Furthermore, hepatitis B envelope antigen (HBeAg) is secreted from infected hepatocytes and cross the blood-placental barrier, leading to the neonatal tolerance to HBV and to the suppression of virus-specific T cell responses [22].

HCC is a highly heterogeneous disease from a molecular viewpoint [23,24]. HCC represents a classic paradigm of inflammation-associated malignancy, since most of the tumors are arising in the context of hepatic inflammation and the resultant fibrosis [25,26,27]. The risk factors of HCC usually provoke a non-resolving inflammatory response characterized by infiltration of lymphocytes, macrophages, and immature myeloid cells. These immune cells produce various kinds of inflammatory cytokines, resulting in the perpetuation of the wound-healing response that ends up with cirrhosis and HCC [25]. Recent immunogenomic analysis by utilizing data compiled by The Cancer Genome Atlas (TCGA) has classified HCC as C4 subtype characterized by enrichment of M2 macrophages and suppression of Th1 CD4^+^ T cell response. These data suggest that tumor microenvironment in HCC is usually dominated by immune regulatory cells and resultant immune suppression [28,29].

In the early hepatocarcinogenesis, the NK cells have critical functions. NK cells are innate lymphocytes that are capable of killing virus-infected or cancer cells [30]. In a c-myc/tgfa transgenic mouse model of aggressive human liver cancer, persistent deregulation of numerous immune-related genes were noted at the early stages of hepatocarcinogenesis. Interestingly, at this stage, hepatic NK cell frequency declined dramatically, although loss of major histocompatibility complex (MHC) class I expression and upregulation of activating ligands for NK cells was noted in dysplastic hepatocytes [31]. These findings suggest that the disruption of immune surveillance by NK cells denotes the onset of HCC development [31].

In this review, we will discuss the mechanisms of NK cell dysfunction in patients with HCC. Then, we will summarize the recent developments and future directions in HCC management by enhancing the functions of endogenous NK cells and by infusing patients with cultured NK cells.

## 2. Biology of Human NK Cells

Although bone marrow is the principal site of NK cell development, they may also develop in thymus and liver. NK cells undergo progressive stages of acquisition of specific receptors, maturation, and expansion [32].

NK cells mediate cytotoxicity and the cytokine-producing effector functions [33]. NK cells are defined as CD3^−^CD56^+^ cells in humans and CD3^−^NK1.1^+^ or CD3^−^NKp46^+^ cells in mice [34]. In humans, two main subsets of NK cells, the CD56^bright^ and the CD56^dim^ NK cells are distinguished by their cell surface CD56 levels. Amongst them, the CD56^dim^ NK cells constitute approximately 90% of the total NK cells, show mature phenotype, and mediate cytolytic reactions. In contrast, the immature CD56^bright^ NK cells constitute 5% to 15% of the total NK cells and have been regarded as cytokine producers [35,36]. The most important cytolytic targets of NK cells are the abnormal cells having the downregulated expression of the MHC class I, which is highly expressed on the surface of most of the healthy, nucleated cells [37]. NK cells can be quickly activated when they encounter virus-infected cells or tumor cells, since the loss of MHC class I is a common mechanism in these cells to avoid recognition by the cytotoxic T cells [38].

Activated NK cells rapidly establish dynamic contacts with the target cells via integrins, such as the leukocyte functional antigen-1, followed by the exocytosis of perforins and granzymes which damage the cell membrane and elicit apoptosis [39]. Death-receptor pathways involving Fas ligand and TNF-related apoptosis-inducing ligand (TRAIL) are also used to kill the target cells. Mature NK cells are not activated when they contact normal cells that express MHC class I molecule, which can bind to inhibitory receptors on the surface of NK cells. The inhibitory receptors recruit tyrosine phosphatase to remove the phosphates from several signaling proteins or lipids that were generated by the activating receptors [39]. When a NK cell comes across abnormal cells lacking the MHC class I, inhibitory receptors are not engaged, and the killing of the target cells is triggered by the unsuppressed activating signals [40]. In this manner, NK cell function is regulated by a dynamic balance between signals produced from the activating and inhibitory receptors [40].

Among the activating receptors of NK cells, FcγRIIIA (CD16) is the only receptor that can individually evoke the signal that is sufficient enough to stimulate degranulation after crosslinking by monoclonal antibody [41]. CD16 binds to the Fc portion of immunoglobulins and transmit activating signals in NK cells [42]. Once activated by Fc receptors through monoclonal antibodies that are bound to target cells, NK cells kill target cells with cytotoxic molecules, and secrete cytokines to recruit the adaptive lymphocytes. This is known as the antibody-dependent cell cytotoxicity (ADCC) function, which is the primary mechanism of action for some monoclonal antibodies [42]. All other activating receptors such as the NK group 2D (NKG2D), natural cytotoxicity receptors (NCRs), DNAX Accessory Molecule-1 (DNAM-1), 2B4, and CD2 can only work in combination with each other [40].

NK cells have not only cytotoxic function but also immunoregulatory roles that can have positive or negative influences on the overall immune responses by modulating the function of T cells and dendritic cells (DCs) [34]. NK cells secrete proinflammatory cytokines and chemokines such as TNF-α, IFN-γ, IL-6, and CCL5 that may contribute to innate and adaptive immune responses [34]. Furthermore, NK cells respond to a variety of cytokines and chemokines from the external environment. Cytokines such as the interleukin (IL)-2, IL-12, IL-15, and IL-18 are strong activators of NK cells. These cytokines have been applied for NK cell-based immunotherapy against various types of cancers [43]. Responding to these cytokines, NK cells secrete cytotoxic molecules such as perforin, granzyme, IFN-γ, TNF-α, and other chemokines and cytokines.

## 3. Intrahepatic NK Cells: Liver-Resident or Liver-Infiltrating

Human liver has a large population of NK cells which form between 30% to 50% of the intrahepatic lymphocytes [44]. This percentage is two to five times the number of peripheral NK cells. In humans, NK cells normally exist in the liver sinusoids [45]. Previous reports have demonstrated that up to 80 percent of intrahepatic NK cells are liver-resident and have a distinct functional and transcriptional signature in humans [44,46,47,48]. These CD56^bright^CD16^−^CD69^+^CXCR6^+^ human liver-resident NK cells survive for a relatively long duration in the liver and are incapable of recirculation, whereas the liver-infiltrating NK cells have the transcriptional characteristics of the peripheral NK cells [46]. This concept of tissue residency was also used to describe tissue-resident memory T (Trm) cells. A recent study using mouse models has demonstrated that the transcription factor Hobit is specifically upregulated in tissue-resident lymphocytes including Trm cells, NKT cells, and liver-resident NK cells [49]. In that study, Hobit and Blimp1 repressed several genes involved in tissue egress, including *Klf2*, *S1pr1*, and *Ccr7* [49,50].

Liver-resident NK cells seem to display memory-like features [51]. A proportion of this subset in the human liver expresses CD49a, and has a narrow killer-cell immunoglobulin-related receptor (KIR) profile that indicates a clonal-like expansion [51]. Although it may not be as specific as memory response by adaptive immune cells, NK cell memory can provoke more rapid and stronger responses to the repeated infections. This memory-like feature of liver-resident NK cells may significantly contribute to the cancer immune-surveillance [15,52,53]. Furthermore, the liver-resident NK cells have been found to have some attributes related to the tolerogenic characteristics of the liver [48,54]. Compared to the NK cells found in peripheral blood, liver-resident NK cells express the inhibitory receptor natural killer group 2 member A (NKG2A), which binds to the human leukocyte antigen (HLA)-E in humans, and MHC class I-associated protein Qa-1 in mice. Tolerogenic immune profile of the liver may partly be influenced by the expression of NKG2A on the surface of intrahepatic NK cells [50,55]. A recent study using mouse model has demonstrated that the absence of NKG2A resulted in the expansion of virus-specific CD8^+^ T cells [50,56]. Another way liver-resident NK cells contribute to intrahepatic tolerance is to eliminate virus-specific CD8^+^ T cells or activated CD4^+^ T cells via TRAIL-mediated pathway during chronic viral infection. Under the circumstances, liver-resident NK cells may elicit negative regulatory functions in antiviral immune responses [21,50,57].

In the liver, NK cells actively interact with other immune cell subsets, hepatocytes, and stellate cells. NKT cells, DCs and Kupffer cells can stimulate the activation of NK cell by producing various cytokines, such as type I interferon (IFN), IFN-γ, IL-2, IL-12, IL-15, and IL-18 [44,55]. For example, Guidotti et al. demonstrated that IFN-γ-induced non-cytopathic antiviral mechanisms by NKT-activated NK cells contributed to viral clearance during acute viral hepatitis in the chimpanzee model [58]. Another study reported that TLR-dependent crosstalk between human Kupffer cells and NK cells activates NK cells through IL-18 [59]. These studies show the possible interaction of human NK cells with other immune cell subsets in the liver, which lead to the activation of NK cells. Activated NK cells attack the cholangiocytes, hepatic stellate cells, and hepatocytes, and carry out a range of essential roles in the pathogenesis of liver diseases [44,55]. However, DCs, Kupffer cells, MDSCs, regulatory T cells (Tregs), and hepatic sinusoidal endothelial cells are known to produce IL-10 and TGF-β to inhibit NK cell function and shape tolerance [44,60].

## 4. NK Cells in Chronic Viral Hepatitis

The tolerogenic properties of the liver make it vulnerable to pathogens and sustained chronic infection. In fact, several widespread pathogens, including HCV and HBV, preferentially attack the liver and cause persistent infections. Co-culture experiments demonstrated that NK cells suppress HCV replications by the production of IFN-γ [61]. Earlier genetic studies on KIRs and HLA in HCV-exposed individuals demonstrated the critical function of NK cells in HCV infection [62]. This study was the first to show that the spontaneous HCV clearance is linked to the KIR2DL3/HLA-C1 genotype [62]. In a study performed in Korea, a lower frequency of KIR2DS2 was reported among patients with chronic HCV infection compared to healthy controls, suggesting that KIR2DS2 might facilitate HCV clearance by enhancing the innate immune response [63].

During chronic HCV infection, NK cells are functionally deviated toward increased cytotoxicity and decreased IFN-γ production, by chronic exposure to type I IFNs [64]. Peripheral blood mononuclear cells from HCV-infected patients were cultured in the presence of IFN-α in vitro, which resulted in the increased expression of CD107a and TRAIL in NK cells, but not IFN-γ [64].

This phenomenon is caused by the increased level of signal transducer and activator of transcription 1 (STAT1), and the preferential phosphorylation of STAT1 over STAT4 in NK cells by type I IFN [65,66]. As a consequence, NK cells display accentuated cytotoxicity and TRAIL upregulation, rather than non-cytolytic IFN-γ production [67,68].

Activated NK cells might suppress the replication of HBV and contribute to HBV clearance during acute HBV infection [69]. However, in chronic HBV infection, NK cells are functionally altered similar to that in chronic HCV infection. In particular, their capacity for IFN-γ and TNF-α production is reduced, while their cytotoxic activity is maintained and the TRAIL, CD38, and Ki-67 expressions are increased [70,71,72]. This deviated NK cell function in chronic HBV infection, suppresses the HBV-specific T-cell function [57]. This “inflammatory” phenotype of the NK cells significantly disappears when the viral DNA titer is reduced after successful antiviral therapy [57]. The functional deviation of NK cells in chronic HBV infection is caused by the immunosuppressive cytokines such as TGF-β and IL-10 [71,73].

Chronic liver diseases finally lead to the deposition of extracellular matrix and fibrosis [45,74]. At this stage, the human intrahepatic NK cells take an anti-fibrotic role by targeting the activated stellate cells, which is the major cell type that deposits the extracellular matrix [74]. Activated hepatic stellate cells express ligands for the activating NKG2D and NKp46 receptor, causing the cells killed by NK cells [75]. However, it is still not clear whether these anti-fibrotic activities are performed by the conventional NK cells or the liver-resident NK cells.

## 5. NK Cell Dysfunction in HCC

NK cells play critical roles in the surveillance and control of HCC. However, NK cells are dysfunctional in the microenvironment of HCC, and various mechanisms seem to be involved in their malfunction. Table 1 summarizes various mechanisms of NK cell dysfunction in HCC.

### 5.1. NK Cell Dysfunction in HCC: Decreased Frequency and Defective Cytokine Secretion

**Figure 1:**
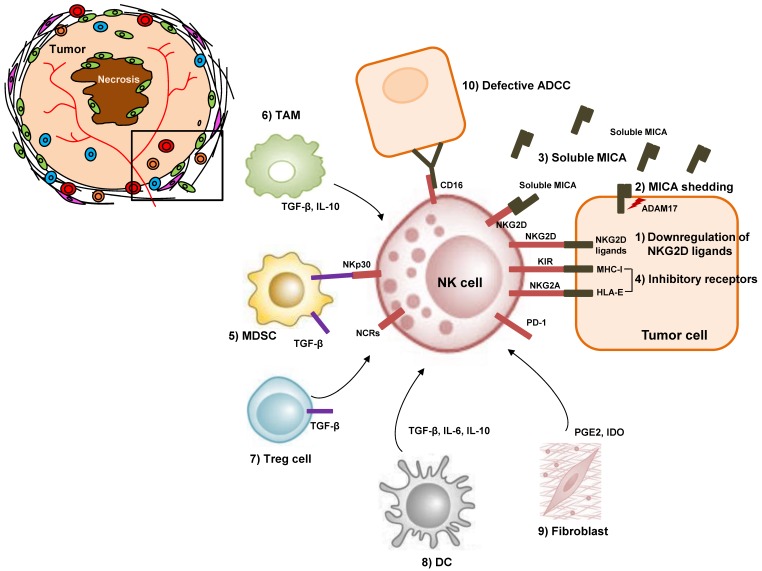
Mechanisms of NK cell dysfunction in tumor microenvironment of HCC. HCC tumor cells inhibit NK cell activity via (**1**) downregulation of NKG2D ligands and (**2**) shedding of membrane-bound MICA. (**3**) Soluble MICA works as a decoy to prevent anticancer surveillance by NK cells. (**4**) Inhibitory receptors such as KIRs and NKG2A on the surface of NK cells recognize their ligands and suppress NK cell activity. (**5**) MDSCs inhibit NK cell cytotoxicity, via membrane bound TGF-β and the NKp30 receptor on NK cells. (**6**) TAMs, (**7**) Tregs, (**8**) DCs, and (**9**) tumor-associated fibroblasts inhibit NK cells via immunosuppressive cytokines. (**10**) Defective ADCC also occur between tumor cells and NK cells in HCC. NK, natural killer cell; HCC, hepatocellular carcinoma; MDSC, myeloid-derived suppressor cell; TAM, tumor-associated macrophage; Treg, regulatory T cell; MHC-I, major histocompatibility complex class 1; TGF-β, transforming growth factor-β; MICA, MHC-I polypeptide-related sequence A; KIR, killer cell immunoglobulin-like receptor; ADCC, antibody-dependent cell cytotoxicity; PGE2, prostaglandin E2; IDO, indoleamine 2,3-dioxygenase; NKG2A, natural killer group 2 member A; NKG2D, natural killer group 2 member D; HLA-E, human leukocyte antigen E; PD-1, programmed death 1; NCR, natural cytotoxicity receptor.

As mentioned earlier, NK cells play a critical role in controlling HCC. High frequency of NK cells with functional activity, expressing a wide spectrum of activating receptors and low amounts of inhibitory receptors, take part in controlling the HCC [76]. In addition, the density of the infiltrating intra-tumoral NK cells is positively correlated with the overall survival in patients with HCC [76,77]. These results imply that NK cells play an important role in the immune-mediated defense against HCC. However, the frequency of NK cells in the tumor regions is lower than that in the non-tumor regions, which would increase the chances of the tumor’s evasion of immune surveillance. Previous report studied the immune profile of 110 patients with HCC and found that NK cells were abundant in the liver tissues of HCC individuals, with higher number of NK cells being detected in non-tumor liver tissues, than in the intra-tumoral regions [78]. Consistent with this finding, a recent report demonstrated that NK cells were infiltrated in the intra-tumoral regions of HCC at a lower frequency than in the non-tumor liver tissues [46]. Most intra-tumoral NK cells exhibited CXCR6^+^ CD69^+^ liver-resident phenotype [46]. In addition, the intra-tumoral NK cell subgroups (CD56^dim or bright^) displayed a distorted population ratio, with a considerable drop in the number of CD56^dim^ NK cells [78]. The functional capacity of intra-tumoral NK cells is also attenuated in the HCC. Figure 1 describes various mechanisms of NK cell dysfunction in the tumor microenvironment of HCC. A previous research revealed that the intra-tumoral NK cells have more defective IFN-γ and TNF-α secretion than non-tumor NK cells [77]. The attenuated cytokine secretion in intra-tumoral NK cells is mainly caused by the defective recognition of tumor cells, or by inhibitory cells surrounding the NK cells, which will be discussed in the following sections. In summary, intra-tumoral NK cells in HCC are functionally defective, with relatively low level of cytotoxic potential and cytokine secretion ability, as compared to the liver-resident NK cells in non-tumor regions.

### 5.2. NK Cell Dysfunction in HCC: Defective Recognition of Tumor

The activating receptors of NK cells are important in tumor immunosurveillance [79]. NKG2D detects several molecules, such as the cytomegalovirus UL-16 protein (ULBP1-6) and polymorphic MHC class I chain-associated molecules (MIC) A/B [79]. These molecules are not usually expressed on the surface of healthy cells, but can be induced in various cell types by stressors such as infection, sterile inflammation, chromatin remodeling, or malignant transformation [40]. The role of NKG2D in HCC has also been emphasized by Chu et al. [80]. These authors found that, at the end of the antiviral therapy, there was a fast down-regulation of NKG2D on peripheral NK cells, in individuals who developed HCC immediately after HCV eradication. One recent study explained that the intra-tumoral NK cells have NKG2D downregulation in comparison to NK cells in non-tumor liver, leading to the defective recognition of tumor [46].

Furthermore, some tumor cells also downregulate NKG2D ligands, or downmodulate NKG2D function on effector cells. Among the NKG2D ligands, MICA was highlighted because the unidentified locus in the 5′ flanking region of MICA was strongly associated with HCC occurrence in HCV-infected patients [81]. Membrane-bound MICA stimulates NK cell-mediated cytotoxicity. However, some proteases in the tumor microenvironment have been reported to shed the membrane-bound MICA, releasing soluble MICA into the bloodstream. According to Jinushi et al., the amount of soluble MICA was high in several patients with HCC, and their respective peripheral NK cells had reduced amounts of NKG2D expression and showed impaired activation [82]. This detrimental function of soluble MICA, enables cancer cells to evade the NK cell-mediated immune surveillance [83]. Among the MICA-shedding proteases, the roles of a disintegrin and metalloproteases 17 (ADAM17), were emphasized recently [83]. ADAM17 knockdown reduced the soluble MICA levels and increased the membrane-bound MICA expression in HCC cell lines, thereby allowing for the cells to be killed by NK cells (Figure 1) [83].

The NCR family is type I transmembrane glycoproteins including NKp30 (NCR3), NKp44 (NCR2) and NKp46 (NCR1) [40]. The *NCR3* gene is transcribed and undergoes alternative splicing, resulting in production of three major isoforms of the NKp30 protein [84]. Recent study demonstrated that NKp30-expressing human NK cells have a reduced expression of NCR3 immunostimulatory splice variants and an increased expression of the inhibitory variant in patients with advanced HCC, leading to defective NKp30-mediated functionality [84]. 

### 5.3. NK Cell Dysfunction in HCC: Role of the KIR-HLA Complex

KIRs are known to inhibit NK cell activation by interacting with diverse MHC class I molecules [85]. The KIR genes, as well as their cognate HLA genes, illustrate extensive polymorphism and produce varied NK cell responses to cancer cells. There are two different points that should be considered in the role of KIR-HLA complex in patients with HCC: the “licensing” of NK cells during maturation, and the control of NK cell activity when stimulated by the activating signal (Figure 1).

The binding of high-affinity inhibitory KIRs to their HLA cognates exerts a strong inhibitory effect on NK cell activation. However, during the maturation of competent NK cells, the ‘licensing’ of NK cells occurs, to avoid attacking the self [86]. The ‘licensing’ refers to a process by which NK cells expressing the inhibitory KIRs for self-HLA, finally obtain a higher resting response capacity. Five inhibitory KIRs and their cognate HLA ligands that cause the NK cell licensing have been defined till now: KIR3DL1 for the HLA-Bw4 group alleles, KIR3DL2 for the HLA-A3/11 alleles, KIR2DL2 and KIR2DL3 for the HLA-C1 group, and KIR2DL1 for the HLA-C2 group of alleles [87,88]. In line with the “licensing” model, an immunogenetic study discovered that the presence of KIR2DL2 and homozygosity for HLA-C1 that conferred NK cell licensing, correlated with the prolonged recurrence-free survival in patients with HCV-related HCC, after radiofrequency ablation [89]. This means that the concurrent presence of KIR2DL2 and HLA-C1 is associated with longer time-to-recurrence, due to the protective effect of NK cells that had attained full functional competence after licensing [89]. However, for patients with HBV infection, the KIR-HLA types with the high functional maturation of NK cells, which had been ‘licensed’, were associated with HCC progression [90]. This can be partly explained by the assumption, that an enhanced phenotype of NK function might lead to tissue damage as well as constant inflammation, which promoted HCC development in patients with chronic HBV infection [91]. However, in allogeneic NK cell therapy, KIR-ligand incompatibility seems to be critical because the mismatch prevents the generation of negative signal and guarantees adequate NK cell activation [30]. Although not fully defined yet, the progression of HCC is somewhat attributed to the dysfunction of NK cells, and the immunogenetic profile of KIR/HLA is linked to the activity of NK cells, which affects the prognosis for patients with HCC.

### 5.4. NK Cell Dysfunction in HCC: Inhibitory Roles of the Immunoregulatory Cells and the Immunosuppressive Cytokines

One mechanism that modulates the function of NK cells is the crosstalk between immune cells in the tumor microenvironment (Figure 1). In the liver, multiple cellular constituents such as MDSCs, Tregs, macrophages polarized to the immunoregulatory phenotype, and immature DCs facilitate the development of cancer by promoting local immune tolerance [92]. Normally, Tregs infiltrate the HCC, and the HCC stages have correlated with their frequencies [93,94]. They impair NK cell responses via membrane-bound and secreted TGF-β [95,96]. Interestingly, Treg frequency gets lower in mice with STAT3-blocked HCC cells, indicating that STAT3 activation in tumor cells may be critical in Treg induction and NK cell suppression [97]. MDSCs are myeloid cells with a potent immunosuppressive activity. They accumulate in the intra.tumoral and stromal lesions of various types of cancer, including HCC [98]. Accumulation of MDSC in HCC was also demonstrated in various mice models of HCC [99]. MDSC-mediated inhibition of NK cells is contact dependent via membrane-bound TGF-β on MDSC [100]. Furthermore, recent data has shown that MDSCs from patients with HCC hamper cytokine production and cytotoxicity by autologous NK cells, and the suppression is cell contact dependent and primarily relies on NKp30 on the surface of NK cells [101]. Tumor-associated macrophages (TAM) are located in the stroma of HCC and are polarized toward M2 phenotype [102]. TAMs foster tumor cell proliferation and spread in the HCC [102]. TAMs are therefore related with the increased recurrence of tumor after the surgical resection of HCC [103]. The primary role of TAM is their cytokine-dependent inhibition of NK cells and other lymphocytes with IL-10 and TGF-β [104]. In addition, the macrophages from intra-tumoral regions of HCC express CD48 proteins, which interacts 2B4 on NK cells and cause NK cell dysfunction [77]. The immunosuppressive activity of TGF-β in the inhibition of NK cell cytotoxicity was also reported when NK cells are co-cultured with DCs [100]. Secretion of IL-6 and IL-10 is also involved in DC-mediated NK cell inhibition [105]. HCC-associated fibroblasts also induce the NK cell dysfunction by producing prostaglandin E2 (PGE2) and indoleamine 2,3-dioxygenase (IDO) [106].

## 6. Strategies to Boost NK Cell Function in the HCC Microenvironment

To reverse the malfunction of NK cells in HCC, various strategies were developed. These approaches include either endogenous stimulation of the NK cells in patients, or adoptive NK cell therapy to the patients. This review will cover both approaches in the following sections and Table 2.

### 6.1. Strategies to Boost NK Cell Function in the HCC Microenvironment: Current Treatment Options

Currently, there are some effective therapies accessible to patients with HCC. The curative therapeutic options for HCC include the liver transplantation, radiofrequency ablation and surgical resection [1,107]. Radiofrequency ablation and surgical resection may have different impacts on the NK cell function. Ohira et al. discovered that the NK cells had a lower expression of CD69 and TRAIL after partial hepatectomy in mice. In addition, after injecting the Hepa 1–6 cell line, these mice were vulnerable to liver metastases in the remaining portion of the liver [108]. This observation needs to be validated in humans. In contrast, the radiofrequency ablation was linked to an evident increase in the IFN-γ secretion and NK cell cytotoxicity [109]. Very recently, transcriptomic analysis and deep immunophenotyping were performed with HCC tissues from patients who underwent Y^90^-radioembolization [110]. The data showed strong immune activation in the tumor microenvironment and in the peripheral blood of patients with HCC with sustained response to Y^90^-radioembolization [110]. Among the immune cells that were recruited and activated, NK cells were the strongest producer of granzyme B, suggesting the critical role of NK cells in the control of tumor, after Y^90^-radioembolization [110]. Sorafenib also enhanced the NK cell-mediated cytotoxicity by increasing the expression of membrane MICA and reducing the levels of soluble MICA from HCC cell lines [111]. Furthermore, through the modulation of the crosstalk between NK cells and TAMs, sorafenib strengthened the cellular anticancer effector functions [102]. In contrast, another group claimed that sorafenib reduces the number and cytotoxicity of NK cells against tumor cells in tumor-bearing mice through downregulation of CD69 on the surface of NK cells [112], which needs further validation. Cisplatin has recently been demonstrated to upregulate an NKG2D ligand, ULBP2, which might consequently enhance the NK cell-induced cytotoxicity against HCC cell lines. This supports a new immunomodulatory role for cisplatin in HCC therapy [113].

### 6.2. Strategies to Boost NK Cell Function in the Microenvironment of HCC: Modulating ADCC and the Function of Activating Receptors

Clinical trials have shown that patients with HCC having a high affinity FcγRIII polymorphic variant have better outcome after monoclonal antibody treatment [42]. This supports the potential advantageous function of ADCC and CD16 in HCC treatment. The ADCC of tumor cells by NK cells has been applied in the treatment of various cancers overexpressing unique antigens [42]. To directly target HCC cells, antibodies targeting the glypican-3 (GPC-3), which is expressed on up to 70% of tumors but not on normal hepatocytes, were prepared and tested [114]. Humanized anti-GPC-3 antibody, codrituzumab, was proven to induce the ADCC. Although the phase I trial for codrituzumab was promising [115], the phase II trial for patients with advanced HCC for whom the standard treatment had failed, did not show an overall survival benefit (NCT01507168) [116]. Subgroup analyses showed that high GPC-3 expression in the tumors and CD16 expression on peripheral NK cells were associated with prolonged overall and progression-free survival [116]. The efficacy of such antibody-based therapies might have been restricted by lower levels of NK cell activation due to the shedding of cell surface CD16, or by CD16 polymorphism [42].

For activating receptors of NK cells other than CD16, the results are slightly complicated. In a recent report, Sheppard et al. showed an unexpected tumor-promoting role of NKG2D in a model of inflammation-driven HCC [117]. They showed that NKG2D-sufficient mice displayed an increased tumor growth associated with an increased infiltration of cytotoxic CD8^+^ T cells to the liver and an intensified pro-inflammatory environment, which finally caused HCC [117]. NKp30, another activating receptor of NK cells, mediated the interaction of human NK cells with the MDSCs in HCC, leading to the inhibition of NK cell activity [101]. Similarly, another activating receptor, NKp46, was shown to be upregulated on peripheral NK cells in HCC patients with a poor prognosis [118]. Collectively, these data suggest that enhancing the function of activating receptors on NK cells might not be beneficial in the control of HCC.

In regard to other aspects of NKG2D signaling, the modulation of NKG2D ligands on the tumor cells can boost the NK cell activity against HCC cells. Soluble MICA, as mentioned earlier, was demonstrated to work as a decoy to block anticancer surveillance mediated by NK cells, while the upregulation of membrane-bound MICA expression enhances the NK-mediated cytotoxicity. Histone deacetylase (HDAC) inhibitors are a new class of anticancer agents for the treatment of various types of cancers including HCC [119]. Several groups have reported that HDAC inhibitors promote MICA or MICB expression on HCC cells and increase the vulnerability of HCC cells to NK cell-mediated lysis [120,121,122]. This suggests that the HDAC inhibitors promote the recognition of tumor cells by immune cells, in addition to their direct role in proliferation inhibition and apoptosis induction in tumor cells [119]. Furthermore, it was recently reported that lomofungin, an antifungal drug, drastically decreases the enzymatic activity of ADAM17 and enhances the membrane MICA expression in a dose-dependent manner [123]. Another recent report demonstrated that the enhancer of zeste homolog 2 (EZH2) functions as a transcriptional suppressor of NKG2D ligands in HCC cells, and targeting of EZH2 by small-molecule inhibitors causes killing of HCC cells by NK cells in an NKG2D ligand-dependent manner [124].

### 6.3. Strategies to Boost NK Cell Function in the Microenvironment of HCC: Modulating the Function of Inhibitory Receptors on NK Cells

Blocking inhibitory receptors can also provide a robust method to enhance NK cell function. A recent clinical trial using the human HLA-haplotype mismatched hematopoietic stem cells to treat leukemia, showed efficient NK cell-mediated elimination of the leukemic cells [125]. This indicates that the unmatched KIR-HLA haplotypes are potent NK cell activators, suggesting a rationale of blocking inhibitory KIR of NK cells to kill tumor cells. The two IgG4 monoclonal antibodies in clinical advancement that target inhibitory NKG2A and KIR2D (such as KIR2DL1-3/S1-2) receptor functions are monalizumab (IPH2201) and lirilumab (IPH2101), respectively [126,127]. Lirilumab infusion resulted in rapid decrease in the surface KIR2D expression and the inhibition of KIR2D^+^ NK cell function, although it did not show clinical efficacy in multiple myeloma [126,127]. The CD94-NKG2A complex provides another inhibitory stimulus, when it interacts with the non-classical MHC-I, HLA-E, on target cells [40]. Monalizumab is an antibody which targets the CD94/NKG2A receptor and improves the cytolytic action of NK cells in pre-clinical investigation. Earlier researches have proposed that HLA-E is differently expressed in HCCs, highlighting the possible use of this monoclonal antibody for HCC therapy [128].

PD-1, which is a well-known exhaustion marker in T cells, was shown to be expressed on the surface of CD56^dim^ NK cells [127]. PD-1-expressing NK cells exhibited defective antitumor action and cytokine-stimulated proliferation, and such characteristics were reversed by blocking the PD-1/PD-L1 activities [129]. Nevertheless, NK-cell contribution to the antitumor action targeting PD-1 axis in HCC remains to be investigated in future research. Another checkpoint molecule, T cell immunoglobulin and immunoreceptor tyrosine-based inhibitory motif domain (TIGIT) was recently found to be highly expressed on the exhausted tumor-infiltrating NK cells [130]. Monoclonal antibody-mediated blockade of TIGIT or combined blockade of TIGIT and PD-L1 boosted the antitumor activity of NK cells in mouse models of colorectal cancer and efficiently delayed tumor growth [130]. TIGIT-mediated NK cell exhaustion needs to be confirmed in human and mouse models of HCC.

### 6.4. Strategies to Boost NK Cell Function in the Microenvironment of HCC: Stimulation of NK Cells with Interferons and Cytokines

After binding of IFNs to their receptors, cellular or extracellular effects are mediated through various interferon-stimulated genes (ISGs) with immunoregulatory and antiviral effects [131,132]. IFNs are grouped into three main categories, namely type I, type II, and type III. Each category of IFNs signals to the host cell by binding to the respective receptor complexes [132,133,134]. An experimental treatment for HCC involving the type I and III IFNs in a BNL hepatoma model showed an important role for NK cells in the antitumor action of IFNs [135]. A phase I clinical trial (NCT01628640) has been initiated for patients with advanced HCC, with a recombinant vesicular stomatitis virus expressing the IFN-β, which may perform an anticancer activity by stimulating the NK cells [136,137].

IL-15 supports the survival, proliferation, and cytolytic activity of NK cells [46,138]. IL-15 is capable of recovering the antitumor function of NK cells hampered by in vitro contact with HCC cell lines or cells taken out directly from HCC tissues [46]. K562 cells transfected with membrane-bound IL-15 and 4-1BB ligand encoding plasmid, can proliferate and activate the NK cells for application in HCC immunotherapy [139]. Continuous infusions of IL-15 in cancer patients is accompanied by a preferential expansion of CD56^bright^ NK cells with increased abilities to recognize tumor cells and with enhanced cytokine production and cytotoxicity [140]. Nevertheless, systemic administration of IL-15 can result in major toxicity, which is associated with IFN-γ secretion by NK cells [141]. Novel approaches to specifically enhance IL-15 signaling in NK cells and reduce the systemic adverse effects will be promising immunotherapy areas to focus on.

## 7. Current Status and Future Direction of Adoptive NK Cell Therapy in HCC

**Figure 2:**
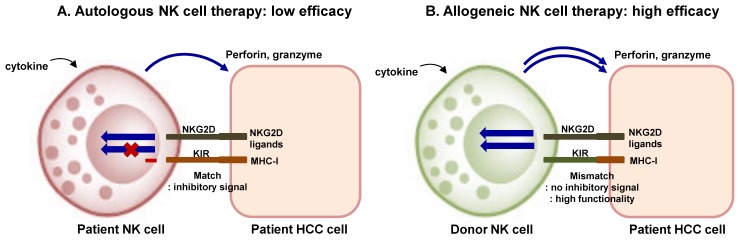
Adoptive transfer of NK cells: Autologous and allogeneic NK cell transfer. (**A**) In autologous NK cell transfer, anti-tumor activity of NK cells might be limited by the inhibitory signal transmitted by the complex of matched KIR and self MHC class I molecule. (**B**) In allogeneic NK cell transfer, high cytotoxic responses can be obtained when donor NK cells do not express KIRs matching the MHC class I molecules of the tumor cells. KIR–ligand incompatibility is critical in efficacy of allogeneic NK cell therapy because the mismatch prevents the generation of negative signal and guarantees adequate NK cell activation. NK, natural killer cell; HCC, hepatocellular carcinoma; KIR, killer cell immunoglobulin-like receptor; NKG2D, natural killer group 2 member D; MHC-I, major histocompatibility complex class 1.

Adoptive therapy of NK cells requires ex vivo expansion, and in vivo specificity to tumor cells, and in vivo maximal activity and persistence [34]. The source of NK cells for adoptive therapy can be autologous or allogeneic NK cells, stem cell-derived NK cells, and NK cell lines such as NK-92 cell line (Figure 2) [9]. Transfer of autologous NK cells failed to show improved survival outcomes in several types of cancers, because these cells had low efficacy and tended to remain in circulation rather than in tumor microenvironment (Figure 2) [142,143]. Therefore, allogeneic NK cell transfer is recently being widely used in various clinical trials. Most cases of allogeneic NK cell trials have been performed using HLA-haploidentical NK cells with or without allogeneic hematopoietic stem cell transplantation (HSCT) [30]. Adoptive transfer of allogeneic NK cells from KIR-mismatched donors has shown promising results in patients with acute myeloid leukemia [34]. KIR-ligand incompatibility seems to be critical in efficacy of allogeneic NK cell therapy because the mismatch prevents the generation of negative signal and guarantees adequate NK cell activation (Figure 2). Allogeneic NK cells are expanded with cytokine stimulation, and T cells should be removed before adoptive transfer to avoid graft-versus-host disease [30].

Recently, the clinical efficacy of the allogeneic NK cell immunotherapy to treat various types of hematologic and solid tumors has also been evaluated by many groups. In HCC, there are on-going clinical trials using adoptive NK cell therapies (Table 2). A Chinese group recently demonstrated that irreversible electroporation combined with allogeneic NK cell transfer significantly increased the median overall survival of patients with stage IV HCC (NCT03008343) [144]. Allogeneic NK cell transfer trial for preventing HCC recurrence after curative resection had been performed in Korea (NCT02008929), although the results are not available. Recently, a Korean multicenter group started a new phase 2 clinical trial to evaluate the safety and efficacy of allogeneic NK cells therapy after trans-arterial chemoembolization (NCT02854839). In another clinical trial, allogeneic, IL-2-stimulated NK cells from liver allografts were intravenously injected to transplant recipients. The safety and feasibility of this therapy was demonstrated in the phase I trial (NCT01147380) [145].

Genetic modification is another option for redirecting the function of NK cells. Genetic manipulation of NK cells has proven to be difficult until recently. Viral transduction was reported to be associated with very low expression levels of inserted genes and harmful effects on cell viability in NK cells. The recent optimization of viral transduction for efficient gene transfection, have been shown to boost the interaction and activities of tumors and NK cells. First, it can be performed to express cytokine transgenes. NK cell function is enhanced by forced expression of cytokine transgenes such as IL-15, IL-2, or IL-12 [30]. Gene therapy with an adenovirus expressing IL-12 against orthotopic HCC in rat model, showed a considerable control of tumor growth. Activation of NK cells was the primary antitumor mechanism involved [146]. Intra-tumoral gene transfer of IL-12 in the mouse HCC model inhibited the spontaneous lung metastasis, neovascularization, and tumor growth [147]. Here again, the tumor growth inhibition was almost completely dependent on NK cells, and this result was confirmed by the depletion of NK cells [147].

Chimeric antigen receptor (CAR)-expressing NK cells represent another promising modality for genetic modification. Currently, 9 clinical trials are being performed to evaluate the safety and efficacy of CAR-NK cells [142]. CAR-NK cells are short-lived, and known to cause cytokine storms or graft-versus host diseases less frequently than do the CAR-T cells [148]. NK-92 cell line has been used as a source for adoptive CAR-NK cell therapy due to its efficiency in expansion and transduction [149]. Very recently, their potential efficacy was demonstrated in a preclinical model of HCC by a Chinese group [150]. GPC3-specific CAR-NK-92 cells showed potent antitumor activities only to HCC cells expressing GPC3 molecule, reflecting the safety and specificity of CAR-NK cells [150].

## 8. Conclusions

The strong antigen-independent cytotoxicity of NK cells can be applied to various types of cancer immunotherapy. The signals required to elicit robust antitumor responses by NK cells have not yet been entirely elucidated. The role of NK cells in controlling HCC has been underrated. NK cells have been demonstrated to play critical roles in the immune responses against HCC, providing a rationale for developing novel treatment strategies that enhance the NK cell response for treating HCC. A more comprehensive understanding of the detailed receptor interactions with tumor cells, intracellular signaling, and interactions with other immune cells should be reached. This would be critical for evaluating the current efforts to establish novel ways to enhance the activity of NK cells against HCC.

## Figures and Tables

**Table 1:** Mechanisms of NK cell dysfunction in HCC.

Mechanism		Evidence	Host	Ref.
Decreased frequency and distorted subpopulations	Decreased frequency	Decline of hepatic NK cell frequency at early stage of hepatocarcinogenesis (c-myc/tgfa transgenic model)	Mouse	[31]
		Low frequency of NK cells in intratumoral regions, compared with non-tumor liver	Human	[46,78]
	Altered distributions of subpopulations	Reduction in the frequency of cytotoxic CD56^dim^ NK population (intratumoral and peripheral)	Human	[78]
Defective recognition of tumor	NKG2D downregulation	Downregulation of NKG2D on peripheral NK cells when HCC developed after HCV eradication Downregulation of NKG2D in intratumoral regions, compared with non-tumor liver	Human	[46,80]
	NKG2D downmodulation by soluble MICA	Association of high soluble MICA level with reduced peripheral NKG2D expression in HCC patients Shedding of MICA by ADAM17 and resultant NK cell dysfunction in a co-culture model	Human	[82,83]
	Expression of NKp30 inhibitory variant	Reduced level of NCR3 immunostimulatory variants and an increased level of inhibitory variant in intratumoral and peripheral NK cells, resulting in deficient NKp30-mediated functionality	Human	[84]
	Defective ADCC	Association of low CD16 expression on peripheral NK cells with poor response to mAb treatment	Human	[116]
Stimulation of inhibitory receptors	KIR-mediated NK inhibition	KIR-ligand mismatch prevents the generation of negative signal in allogeneic NK cell transfer	Human	[30,34]
	KIR-HLA-mediated NK licensing (maturation)	Association of matched KIR2DL2 and HLA-C1 and delayed recurrence after RFA (HCV-HCC) Association of matched KIR-HLA types and HCC development (HBV-HCC)	Human	[89] (HCV) [90] (HBV)
	NKG2A	Expression of HLA-E, an NKG2A ligand, suggesting the possible inhibitory role of NKG2A in human HCC	Human	[128]
Immunoregulatory cells and the immunosuppressive cytokines	Regulatory T cells (Treg)	Inhibits NK cells via membrane-bound and secreted TGF-β Inhibits NK cells via secreted IL-10 and TGF-β in STAT3-activated HCC	Human Mouse	[95,96] (Human) [97] (Mouse)
	Myeloid-derived suppressor cells (MDSC)	Accumulation of MDSC in mice with HCC irrespective of the mouse models Inhibits NK cells via membrane bound TGF-β and the NKp30 receptor on NK cells	Mouse Human	[99] (Mouse) [101] (Human)
	Tumor-associated macrophages (TAM)	Deviated to immunoregulatory M2 phenotype Inhibits NK cells via IL-10 and TGF-β Expresses CD48, and interacts with 2B4 on NK cells, causing NK cell dysfunction	Human	[77,102]
	Immature DC	Inhibits NK cells via secretion of IL-6 and IL-10	Human	[105]
	Fibroblasts	Inhibits NK cells via PGE2 and IDO	Human	[106]

NK, natural killer cell; HCC, hepatocellular carcinoma; NKG2D, natural killer group 2 member D; MICA/B, MHC-I polypeptide-related sequence A/B; ADAM17, a disintegrin and metalloproteases 17; NCR3, natural cytotoxicity receptor 3; ADCC, antibody-dependent cell cytotoxicity; KIR, killer cell immunoglobulin-like receptor; HLA, human leukocyte antigen; HCV, hepatitis C virus; HBV, hepatitis B virus; NKG2A, natural killer group 2 member A; TGF-β, transforming growth factor-β; PGE2, prostaglandin E2; IDO, indoleamine 2,3-dioxygenase; Ref, reference.

**Table 2:** Strategies to overcome NK cell dysfunction in HCC.

Strategies		Potential mechanism/Features	Development Stage	Country	ClinicalTrials.gov Identifier	Ref.
Current treatment options	Radiofrequency ablation	May enhance the NK cell-mediated cytotoxicity	Current use			[86]
	Y^90^-radioembolization	May enhance the NK cell-mediated cytotoxicity	Current use			[87]
	Sorafenib	May enhance the NK cell-mediated cytotoxicity	Current use			[88]
	Cisplatin	Upregulates an NKG2D ligand in HCC cells	Current use			[90]
Monoclonal antibodies	Codrituzumab (anti-GPC-3-antibody)	Induces ADCC of tumor cells expressing GPC-3 No survival benefit	Phase II	Multinational	NCT01507168	[93]
Upregulation of NKG2D ligands	HDAC inhibitors	Promote MICA/B expression on HCC cells	Preclinical			[118,119,120]
	Lomofungin	Decreases the enzymatic activity of ADAM17 and enhances the membrane MICA expression	Preclinical			[121]
Blocking NK inhibitory receptors	Monalizumab (IPH2201)	Antibody targeting the CD94/NKG2A receptor Not studied in HCC				[124]
	Lirilumab (IPH2101)	Antibody targeting the KIR2D^+^ NK cells Not studied in HCC No clinical efficacy in multiple myeloma				[125]
Cytokines	Recombinant vesicular stomatitis virus expressing the IFN-β	For patients with sorafenib-refractory or -intolerant HCC	Phase I	USA	NCT01628640	[134,135]
	Infusion of recombinant IL-15	Preferential expansion of CD56^bright^ NK cells Not tried in HCC patients				[138]
Adoptive cell therapy	Allogeneic NK cells	Performed after transarterial chemoembolization	Phase II	Korea	NCT02854839	
	Allogeneic NK cells	For stage IV HCC patients Combined with irreversible electroporation Survival benefit demonstrated	Phase I, II	China	NCT03008343	[126]
	Allogeneic NK cells	NK cells from liver allografts	Phase I	USA	NCT01147380	[127]
	Autologous NK cells	Low efficacy due to the inhibitory signal from KIR and MHC class I				[140,141]
	CAR-NK cells (GPC3-specific NK-92 cells)	Safe and short-lived Less cytokine storm than CAR-T cells	Preclinical			[148]

NK, natural killer cell; HCC, hepatocellular carcinoma; GPC-3, glypican-3; ADCC, antibody-dependent cell cytotoxicity; NKG2D, natural killer group 2 member D; HDAC, histone deacetylase; MICA/B, MHC-I polypeptide-related sequence A/B; ADAM17, a disintegrin and metalloproteases 17; KIR, killer cell immunoglobulin-like receptor; IFN, interferon; CAR, chimeric antigen receptor; Ref, reference.
